# Claustrum Involvement in New Onset Refractory Status Epilepticus: A Systematic Review

**DOI:** 10.1002/acn3.70490

**Published:** 2026-08-04

**Authors:** Margherita Burani, Lorenzo Muccioli, Giada Giovannini, Niccolò Orlandi, Francesca Bisulli, Stefano Meletti

**Affiliations:** ^1^ Department of Biomedical, Metabolic and Neural Sciences University of Modena and Reggio Emilia Modena Italy; ^2^ Neurophysiology Unit and Epilepsy Centre, Neuroscience Department Modena AOU Modena Italy; ^3^ IRCCS Istituto delle Scienze Neurologiche di Bologna (Full Member of the ERN EpiCARE) Bologna Italy; ^4^ Department of Biomedical and Neuromotor Sciences University of Bologna Bologna Italy; ^5^ Department of Medicine and Surgery University of Parma Parma Italy

**Keywords:** claustrum, epilepsy, FIRES, neuroimaging, NORSE

## Abstract

The claustrum sign is a distinctive neuroimaging finding characterized by bilateral T2/FLAIR hyperintensity of the claustrum, one of the most interconnected regions of the human brain. It was first described in new‐onset refractory status epilepticus (NORSE) and febrile infection–related epilepsy syndrome (FIRES). A systematic search was conducted according to PRISMA guidelines from 2018 (formal definition of NORSE) to February 2026 (PROSPERO 2026 CRD420261307306). We included peer‐reviewed original studies reporting MRI findings and patient‐level data, and compared clinical characteristics, treatments, and outcomes of patients with claustrum alteration versus NORSE/FIRES patients without this finding. Of 785 records identified, 65 studies met inclusion criteria; 41 provided individual patient data, yielding 206 NORSE cases (72 with the claustrum sign, 134 without). All but one patient with the claustrum sign met criteria for cryptogenic NORSE. Patients were predominantly young (mean age 24.9 years) and had a febrile prodrome in 93% of cases. Convulsive status epilepticus (SE) occurred in 98%, progressing to super‐refractory SE (SRSE) in more than half reported cases. Bilateral claustrum hyperintensity was detected in 68/71 patients during the acute phase, typically emerging 7 days after SE onset. Immunotherapy was administered in 92% of patients. In‐hospital mortality was 9%, while 74% of patients developed chronic epilepsy. Compared with patients without claustrum involvement, those with the claustrum sign more often had febrile onset (93% vs. 51%, *p* < 0.001) and convulsive SE (98% vs. 81.5%, *p* = 0.008), lower rates of SRSE (51% vs. 96.5%, *p* < 0.001), and ICU admission (71% vs. 99%, *p* < 0.001), and a trend toward lower mortality (9% vs. 16%, *p* = 0.240). Recognition of the claustrum sign may support early diagnosis and targeted treatment.

**Trial Registration:** PROSPERO 2026 [CRD420261307306]

## 1 Introduction

Status epilepticus (SE) represents the second most frequent neurological emergency in terms of incidence, with variable and potentially severe outcomes [1]. In nearly half of cases, SE is refractory (RSE) to first‐ and second‐line treatments [2]. New‐onset refractory status epilepticus (NORSE) is a form of RSE occurring in previously healthy individuals, without clear etiology identified within the first 72 h [3]. Febrile infection related epilepsy syndrome (FIRES) is a subcategory of NORSE, applicable for all ages, that requires a prior febrile infection starting between two weeks and twenty‐four hours prior to onset of RSE [3]. Although rare, these conditions are devastating and frequently associated with poor prognosis, cognitive decline, and the development of post‐SE epilepsy that is often drug‐resistant [4, 5]. Over the past decades, research has focused on the pathophysiology and etiology of NORSE, which appears to represent a syndrome with distinct biological features, including sustained neuroinflammation, dysregulation of the autoimmune response, excessive cytokine release, and neuronal injury [6, 7, 8]. Furthermore, neuroimaging studies have revealed brain cortical/subcortical alterations that can be observed in the first few days following NORSE onset [9, 10]. These MRI‐detected lesions may progress to atrophy, supporting the concept of acquired brain injury related to SE [11, 12]. Conversely, other lesions appear to be reversible, such as the T2/FLAIR hyperintensity of the claustrum, firstly described in patients with RSE after febrile illness in 2015 [13] and known as the claustrum sign.

The claustrum is a thin, sheet‐like neural structure located between the insular cortex and the striatum. Its organized projections to the entire cortical mantle make the claustrum one of the most extensively interconnected brain regions [14]. Importantly, the primary input to the claustrum originates from limbic, associative, and motor areas of frontal cortex, with fewer inputs from sensory areas. It also receives one‐way inputs from subcortical regions, including the thalamus, basolateral amygdala, and hippocampus, along with neuromodulation from serotonergic neurons of the dorsal raphe nucleus. Its activation strongly drives inhibitory interneurons of cortical pyramidal cells. This feedforward inhibition makes the claustrum central to the seizure network, limiting excessive firing in neural networks and synchronizing spike timing [15]. The claustrum has been implicated in several functions, including amplification of cortical oscillations, attentional allocation, consciousness, and cognition [16].

The aim of this study is to conduct a systematic review of the articles reporting MRI findings in NORSE/FIRES, focusing on the clinical features and outcomes of cases with the claustrum sign and comparing them with those without this MRI finding. Given the lack of established MRI prognostic markers in NORSE/FIRES, we evaluated the potential clinical relevance of the claustrum sign for disease characterization and outcome assessment.

## 2 Methods

The study protocol was registered on the international prospective systematic reviews PROSPERO 2026 CRD420261307306.

The systematic review of the literature was conducted in accordance with the Preferred Reporting Items for Systematic Reviews and Meta‐Analyses (PRISMA) guidelines [17].

The main purpose of the review was to evaluate: (i) the consistency of clinical and radiological course of patients with diagnosis of NORSE or FIRES with T2/FLAIR hyperintensity of the claustrum (claustrum sign); (ii) anti‐seizure medications (ASMs) and immunotherapies response; (iii) clinical outcomes in terms of short‐time mortality and post‐SE epilepsy. Finally, (iv) we compared clinical outcomes in NORSE/FIRES patients with vs. without the claustrum sign.

Our search question was formulated using the Participant, Intervention, Comparison, Outcome (PICO) framework [18] as follows: P: patients with NORSE/FIRES diagnosis with the claustrum sign, I: MRI evaluation, C: patients without the claustrum sign, O: survival and post‐SE epilepsy.

To be consistent with the current definition, the literature search was conducted from 2018, after the first consensus definitions for NORSE and its sub‐type FIRES by an international group of experts [3]. These definitions followed the first description of the claustrum sign in 2015 [13] and the subsequent characterization of its clinical and radiological features in 2017 [19]. The search strategy is detailed in eMethods in Supporting Information.

Data extracted included patient demographics (sex and age at SE onset), prodromal symptoms, semiology of SE, treatment responsiveness, admission to the ICU, need for anesthesia, use of immunotherapy, MRI findings and outcomes in terms of survival and chronic epilepsy. Treatment responsiveness is defined as SE cessation after first‐line therapy with benzodiazepines alone or followed by second‐line treatment with ASMs. RSE was considered a failure of first‐line therapy with benzodiazepines and one second‐line treatment with ASMs. Super‐refractory SE (SRSE) was a status that continued or recurred despite the use of anesthetics for longer than 24 h [20].

We included peer‐reviewed studies with accessible abstracts written in English describing cases with a diagnosis of RSE and reporting MRI findings during/after NORSE including claustrum alteration, as well as any other cortical/subcortical imaging alteration. Studies lacking per‐patient information were excluded. Title and abstract screening and extraction, followed by full‐text review, were independently performed in a blinded manner by two reviewers with expertise in status epilepticus and neuroimaging (S.M. and G.G.). Inter‐reviewer agreement was assessed qualitatively and any disagreement during the full‐text review and data extraction phases was resolved by a third reviewer (M.B.). From each included publication, we extracted the total number of reported patients and collected information about their demographics, clinical features, diagnostic findings, therapeutic interventions, and clinical outcomes.

The risk of bias of the included studies was evaluated using the QUADAS‐2 tool [21], evaluating the domains of patient selection, index test, reference standard, and flow and timing. Each domain was rated as having low, high, or unclear risk of bias, with disagreements resolved by consensus.

SPSS for Windows, version 21 (SPSS Inc., Chicago, IL, USA) was used to analyze all statistical data. Continuous variables were summarized by medians and interquartile ranges (IQRs), and categorical variables were summarized by counts and proportions. All comparisons were performed using a univariate approach; χ2 test or Mann–Whitney U test was used, as appropriate, to analyze demographic and clinical characteristics independently. A *p* < 0.05 was considered statistically significant. Given the limited sample size and the heterogeneity of the included cases, no multivariable analyses were performed. Therefore, the analysis should be considered exploratory.

## 3 Results

**FIGURE 1:**
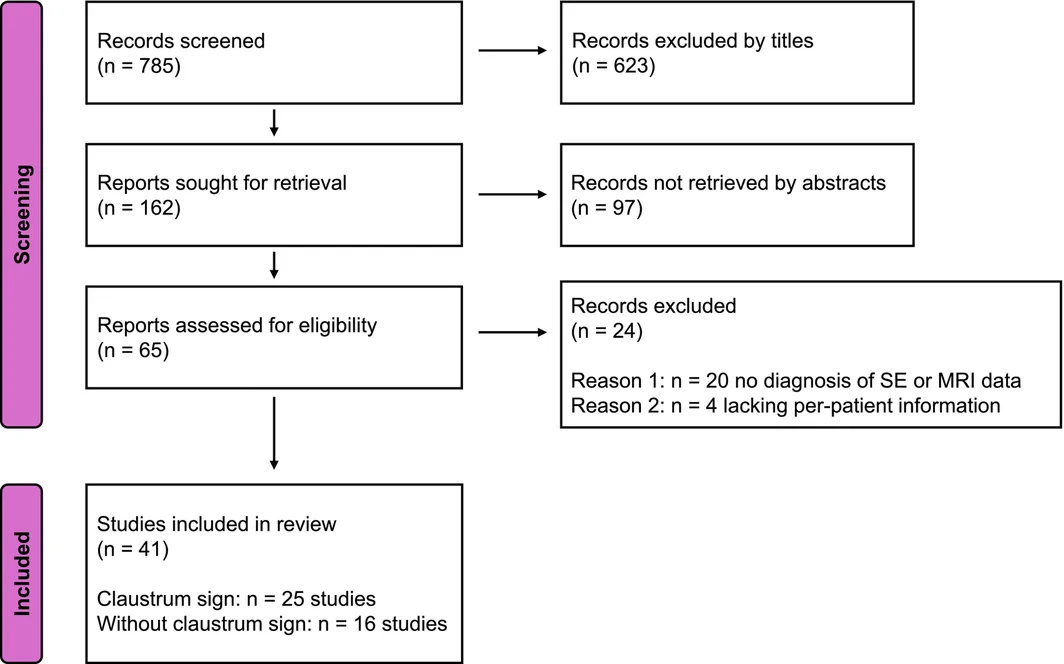
PRISMA flow‐chart.

The initial database search yielded 785 relevant records; after removing duplicates, 162 abstracts were assessed for eligibility and reviewed in full text, and 65 met the predefined inclusion criteria for evidence synthesis (PRISMA flow diagram, Figure 1). Twenty‐four of the selected studies did not provide diagnosis and MRI information or data at the individual patient level and were therefore not suitable. The remaining 41 publications consisted of 25 single case reports and 16 case series, comprising a total of 206 patients: 72 patients with the claustrum sign and 134 patients without claustrum involvement. Clinical, treatment, and outcome data of the two groups are reported in Table 1. The geographical distribution of the publications and of the included cases is shown in Supporting Information: eFigure1.

**TABLE 1:** NORSE patients with and without claustrum sign.

	With claustrum sign (*n* = 71)	Without claustrum sign (*n* = 134)	*p*
Median age, years [IQR]	23 [10.0–36.0]	20 [9.7–33.2]	0.354
Female, *n* (%)	38 (53%)	69 (51%)	0.782
Median time from symptoms to SE onset, days [IQR]	6 [5.0–7.0]	5 [4.0–7.0]	0.150
Symptoms, *n*/total (%)			
Fever only	26/71 (37%)	42/134 (31%)	< 0.001
Fever and others	40/71 (56%)	27/134 (20%)	
Other than fever	3/71 (4%)	2/134 (2%)	
None or not reported	2/71 (3%)	63/134 (47%)	
Type of SE *n*/total (%)			
GCSE	39/54 (72%)	45/124 (36%)	< 0.001
Focal evolving into bilateral convulsive SE	9/54 (17%)	38/124 (31%)	
Focal motor SE	4/54 (7%)	18/124 (14%)	
Myoclonic SE	1/54 (2%)	0/124 (0%)	
NCSE	1/54 (2%)	23/124 (18%)	
Type of SE *n*/total (%)			
Motor SE	53/54 (98%)	101/124 (81.5%)	0.008
NCSE	1/54 (2%)	23/124 (18.5%)	
Need for ICU, n/total (%)	37/52 (71%)	73/74 (99%)	< 0.001
Responsiveness of SE, *n*/total (%)			
RSE	21/43 (49%)	2/57 (3.5%)	< 0.001
SRSE	22/43 (51%)	55/57 (96.5%)	
Immunotherapy, *n*/total (%)	61/66 (92%)	59/66 (89%)	0.545
First line	37/58 (64%)	33/47 (70%)	0.488
Second line	21/58 (36%)	14/47 (30%)	
In‐hospital survival, *n*/total (%)	65/71 (91%)	113/134 (84%)	0.240
Epilepsy, *n*/total (%)	40/54 (74%)	64/99 (65%)	0.232

Abbreviations: GCSE, generalized convulsive status epilepticus; ICU, intensive care unit; IQR, interquartile range; NCSE, non‐convulsive status epilepticus; RSE, refractory status epilepticus; SE, status epilepticus; SRSE, super refractory status epilepticus.

The overall risk of selection bias was assigned as “high” in every study, as they were based on random, non‐consecutive samples, did not use a case–control design and it was not established whether inappropriate exclusions could be avoided by performing diagnostic EEG and MRI according to a predefined timeline and using second‐ and third‐line treatments following standardized guidelines. Assessment bias was not explicitly reported in any of the included studies; however, we estimate it to be high as MRI findings were interpreted by local radiologists and/or neurologists, without any standardized protocol, clinical data were reported differently and inconsistently, and follow‐up data were poor.

### 3.1 Claustrum Sign Patients

Our analysis primarily focuses on the 72 patients presenting with the claustrum sign [22, 23, 24, 25, 26, 27, 28, 29, 30, 31, 32, 33, 34, 35, 36, 37, 38, 39, 40, 41, 42, 43, 44, 45, 46]. 71 patients had negative serum and CSF test results for neurotropic viruses and autoantibodies panel and their etiology remains unknown, fulfilling the definition of cryptogenic NORSE [47]. We excluded one patient [37] who had high titres of anti‐Ma2 antibodies demonstrated in CSF and serum and was diagnosed with autoimmune encephalitis. Detailed clinical characteristics of the definitive claustrum sign group are detailed in Supporting Information: eTable1. 53% of patients (38 out of 71) were female. The mean age at SE onset was 24.9 years (median 23, IQR 10–36).

#### 3.1.1 Prodromal Phase

In 66 patients (93%), fever was the presenting symptom. It was isolated in 26 cases, whereas in the remaining patients it was associated with seizures (*n* = 28), impaired awareness (*n* = 16), psychosis and headache (*n* = 9 each), memory impairment and language disturbances (*n* = 8 each), behavioral changes, drowsiness, confusion, and myalgia (*n* = 6 each), respiratory symptoms (*n* = 5), gastrointestinal discomfort (*n* = 4), hallucinations (*n* = 2) and anorexia in a single case. Prodromal symptoms preceded the onset of status epilepticus by a mean of 6.55 days (median 6, IQR 5–7). For all these reported symptoms, no EEG correlate was available. Therefore, it cannot be excluded that subtle seizures were already present during the “prodromal phase”.

#### 3.1.2 Acute Phase

Patients presented as convulsive SE in 89% of reported cases (39 generalized convulsive status epilepticus [GCSE] and 9 focal onset evolving into bilateral convulsive SE). Four patients developed focal motor SE, one patient presented with myoclonic SE and one with non‐convulsive status epilepticus (NCSE). Information about SE semiology is missing in the remaining 17 cases. Regarding anti‐seizure medications (ASMs), specific treatment data were available for only 21 patients. Among them, 15 received three or more ASMs; 6 were treated with two agents. 21 out of 43 subjects (49%) rapidly developed a RSE, and the remaining 22 (51%) a SRSE, requiring admission to the ICU for 37 patients and anesthesia administration for 25 of them soon after initial presentation.

#### 3.1.3 MRI Findings

Among the 69 patients who underwent brain MRI during the acute phase of status epilepticus, 68 showed bilateral claustrum hyperintensity on T2‐weighted images. On average, the claustrum sign was detected 7 days after SE onset (range 1–20 days), although this information is missing for 21 patients. Bai et al. 2024 provided only aggregate MRI data, reporting a median interval of 12.5 days between SE onset and MRI detection of the claustrum sign.

The only patient with a negative MRI during the acute phase subsequently demonstrated the claustrum sign 25 days after SE onset and approximately two weeks after SE resolution. The two patients who did not undergo MRI during the acute phase, but only after SE resolution, exhibited the claustrum sign at 14 and 15 days from SE onset, respectively.

The bilateral claustrum hyperintensity was isolated to the claustrum in 17 patients (24% of the cases), while in the majority of the cases it was part of a more extended signal hyperintensity alteration including other limbic areas: the medial temporal lobe (*n* = 38), the insula (*n* = 6), the insula and medial‐temporal lobe at the same time (*n* = 5), or the insula and the anterior cingulate gyrus (*n* = 1), the pulvinar (*n* = 2), the basal ganglia (*n* = 2), and other more diffuse cortical areas such as frontal, parietal and occipital lobe (7, 4 and 2 cases respectively). In two cases this sign was seen together with head of caudate hyperintensity and in one case with leptomeningeal enhancement.

Follow‐up MRI study was obtained at various points after resolution of SE in only 45 patients (63%), showing complete resolution in only six cases and bilateral temporal lobe cortical atrophy in five other cases.

#### 3.1.4 Immunotherapy

Besides ASMs and anesthetic therapy, 61 out of 66 patients (92%) received immunotherapy (for five cases this data is missing). High dose intravenous steroids were used in every patient; intravenous immune‐globulins were used in 49 out of 64 patients (76%), and plasma‐exchange in 22% of them. Other immunomodulatory agents such as cyclophosphamide and mycophenolate were used in 4 and 2 cases respectively, while biologic agents were administered in 20 patients (tocilizumab = 10, rituximab = 7 and anakinra = 3). It is important to note that, in most of the included studies, the treatment timeline and the electroclinical response to each therapy were not reported.

#### 3.1.5 Chronic Phase and Outcomes

Five patients died during their initial hospitalization (9%), whereas one patient evolved into a persistent vegetative state, with no subsequent recovery of consciousness. Forty out of 54 patients for whom follow‐up information is provided (74%) developed chronic epilepsy, mostly resistant to medical therapy. Moreover, 16 patients developed cognitive deficits. These deficits were reported to be memory loss (43%), language disturbances (6.7%), executive, and attention deficit (33%). Only 12 patients returned to clinical baseline condition.

### 3.2 NORSE Cases Without the Claustrum Sign

This cohort consisted of 134 patients with a median age of approximately 20 years, 51% of whom were female, presenting with onset of refractory status epilepticus about 5 days after the appearance of initial symptoms (see Table 1). Their brain MRI findings included: 64 patients with no alterations, 45 with medial temporal lobe FLAIR hyperintensity, 14 with hippocampal atrophy, 14 with leptomeningeal enhancement, 12 with basal ganglia and/or insular FLAIR hyperintensity, 4 with diffuse cortical and subcortical alterations, and 2 with corpus callosum FLAIR hyperintensity. Supporting Information: eTable2 reports detailed imaging findings of this group.

### 3.3 Comparison of NORSE With and Without the Claustrum Sign

Table 1 summarizes the comparison between the two groups. NORSE cases with/without the claustrum sign were comparable in terms of median age, sex, and duration of symptoms prior to SE onset. Regarding prodromal symptoms, patients with the claustrum sign presented with fever more frequently (93% vs. 51%, *p* < 0.001). Furthermore, patients with the claustrum sign experienced almost exclusively convulsive SE (98%), most commonly GCSE (72%) and focal onset evolving into bilateral convulsive SE (17%); only a single case was classified as NCSE. In contrast, patients without claustrum involvement presented with NCSE in 18.5% of cases and Motor SE in 81.5% (*p* = 0.008). To note, the number of SRSE was higher in the non‐claustrum group (96.5% vs. 51%, *p* < 0.001) as well as the number of patients admitted to the ICU (99% vs. 71%, *p* < 0.001); conversely, patients with the claustrum sign experienced more RSE responsive to anesthetics (47% vs. 3.5%, *p* < 0.001). Considering immunotherapy, the need for second line agents was similar between the two groups (36% vs. 30%, *p* = 0.488). Finally, patients with claustrum involvement exhibited a non‐significant trend for lower in‐hospital mortality (9% vs. 16%, *p* = 0.240).

## 4 Discussion

In this systematic review, we synthesize the profile of 71 patients, predominantly young individuals, from 25 published reports fulfilling the definition of cryptogenic NORSE, with the claustrum sign. Several key findings emerge from our synthesis:

- Patients with the claustrum sign experienced most of the time convulsive SE with a preceding febrile illness (FIRES);
- The claustrum sign is consistently bilateral and typically appears in the acute/subacute phase of status epilepticus;
- It frequently coexists with limbic and neocortical involvement, suggesting widespread network dysfunction;
- Finally, compared with NORSE patients without claustrum involvement, fewer patients progress to a SRSE and are admitted to the ICU and have a trend for lower in‐hospital mortality, despite a similar higher rate of chronic epilepsy, frequently drug‐resistant.

### 4.1 Radiologic Evolution and Networks Involvement

The claustrum sign was detected during ongoing SE in 68 of 69 patients, emerging on average 7 days after SE onset, with few cases demonstrating delayed detection up to 20–25 days. Notably, in several patients the sign persisted even after SE resolution, reinforcing the concept that it may represent also a subacute or evolving radiologic marker. Follow‐up imaging, although limited, showed complete resolution in only a minority of patients (6/45), and five subjects developed bilateral temporal lobe atrophy. This observation suggests that the claustrum sign may either reflect reversible inflammatory dysfunction or enduring structural network injury, potentially contributing to epileptogenesis [15].

To note, although isolated claustrum hyperintensity was observed in 24% of cases, the majority of patients exhibited additional involvement of limbic and paralimbic regions, particularly the medial temporal lobe and insula. In some cases, more widespread cortical and subcortical regions—including the pulvinar and caudate head—were affected. This pattern strongly supports the hypothesis that the claustrum sign reflects extensive network dysfunction rather than focal structural injury [16].

The claustrum, as a densely interconnected structure, plays a critical role in regulating widespread cortical synchronization and limiting propagation of excessive neuronal firing through feedforward inhibition [14, 15]. Its dysfunction/damage has been proposed to contribute to seizure perpetuation, impaired consciousness, and widespread bilateral cortical networks instability [48]. This hypothesis is also consistent with the rapid development of generalized convulsive SE. Indeed, compared to non‐claustrum patients, subjects with the claustrum sign more typically presented with bilateral convulsive seizures.

### 4.2 Inflammatory and Autoimmune Mechanisms

Nearly all patients with the claustrum are cryptogenic NORSE, with a high prevalence of prodromal fever (93%) indicating that most also fulfilled criteria for FIRES. Current evidence indicates that cryptogenic NORSE/FIRES is characterized by dysregulation of innate immunity and cytokine‐mediated neuroinflammation [49]. Accordingly, the frequent use of corticosteroids, intravenous immunoglobulins, plasma exchange, and escalation to targeted biologic agents (including IL‐6 and IL‐1 pathway inhibitors and B‐cell–depleting therapies) reflects the prevailing clinical perception of immune‐mediated pathophysiology. To note, the claustrum sign has been reported also in other conditions characterized by exaggerated autoinflammatory responses, such as acute necrotizing encephalopathy [50], COVID‐19–related encephalopathy [51], chimeric antigen receptor (CAR) T cell therapy neurotoxicity [52] and other hyperinflammatory states [53], supporting the concept that the claustrum sign may represent a radiologic correlate of cytokine‐mediated neuroinflammation and network dysfunction.

Clinical improvement in some patients following immunomodulatory therapy indirectly supports an inflammatory substrate, although causality cannot be inferred; moreover, the available data on treatment response are reported only as overall clinical outcome rather than as improvement after each therapy.

**FIGURE 2:**
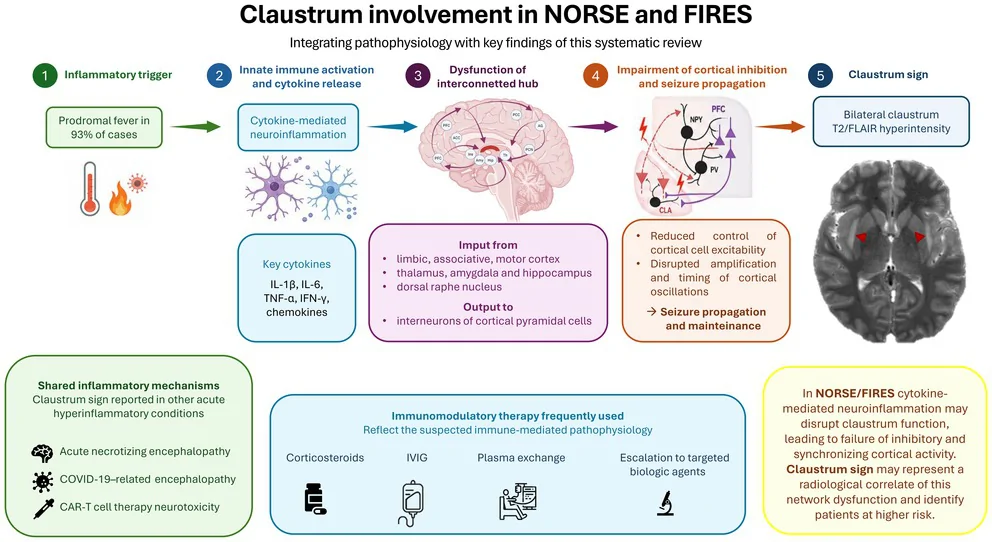
Features of claustrum involvement in new‐onset refractory status epilepticus (NORSE). ACC, anterior cingulate cortex; AG, angular gyrus; Amy, amygdala; CAR, chimeric antigen receptor; CLA, claustrum; FIRES, febrile infection‐related epilepsy syndrome; Hip, hippocampus; Ins, insula; IVIG, intravenous immunoglobulin; NORSE, new‐onset refractory status epilepticus; NPY, neuropeptide Y inhibitory interneurons; PCN, posterior cingulate; PFC, prefrontal cortex; PV, parvalbumin interneurons; Th, thalamus.

Figure 2 integrates the relationship between cytokine‐mediated neuroinflammation, claustrum dysfunction, and seizure network instability in NORSE/FIRES.

### 4.3 Clinical Outcomes and Long‐Term Sequelae

Patients with claustrum sign showed a lower rate of ICU admissions, SRSE, and a higher rate of response to a first cycle of intravenous anesthetics. These differences, however, must be considered cautiously, due to the high risk of bias and to the difference in SE semiology (higher prevalence of NCSE in coma in the non‐claustrum group) [54]. Moreover, for some case‐series, inclusion criteria and fulfilling NORSE criteria can be debated [33]. Mortality during the acute phase was 9%, with one additional patient remaining in a persistent vegetative state, while in the non‐claustrum group mortality occurred for 16% of subjects. We need again to consider this finding in the light of the different rate of convulsive SE and NCSE, because the latter are known to be associated with worse outcomes [55]. Considering long‐term outcomes, follow‐up data were inconsistently available across studies, limiting the reliability of outcome interpretation. Among the 54 patients with available follow up, over two‐thirds of patients developed chronic epilepsy, often drug‐resistant, and 50% exhibited persistent cognitive deficits, most commonly affecting memory and executive function. Only 12 patients returned to their clinical baseline.

These findings reinforce the concept that NORSE associated with the claustrum sign is a condition with substantial long‐term neurological burden. The association between this imaging marker and chronic epilepsy warrants further investigation, as it may have important prognostic implications.

### 4.4 Study Limitations

Our findings must be interpreted considering important limitations. First, the analyses were restricted to a univariate approach, and no multivariable models were performed due to the limited sample size and heterogeneity of the included cases. Therefore, the findings should be considered exploratory and interpreted with caution. For selection bias, it is important to note that all included studies were case reports (*n* = 25) or case series (*n* = 16) and did not adopt a case–control design. Furthermore, patients were not prospectively enrolled according to predefined inclusion criteria. In addition, brain MRI was not standardized across studies and was acquired at different time points, raising the possibility of further selection bias related to imaging acquisition. There is also a risk of assessment bias regarding the interpretation of MRI findings, based on the expertise of local radiologists and/or neurologists. In addition, individual patient‐level data were incomplete in some reports, and long‐term follow‐up was inconsistently documented. All these factors may affect the reliability of the association between the claustrum sign and clinical presentation, and ever more any prognostic considerations. Finally, this systematic review included only reports after the formal consensus definition of NORSE/FIRES established in 2018 [3].

## 5 Conclusions

This is the first systematic review of cryptogenic NORSE/FIRES cases associated with the claustrum sign following the formal definition of these two diagnostic entities [3, 12, 46]. The claustrum sign emerges as a reproducible, time‐dependent neuroimaging marker in a distinct subset of patients with NORSE, typically characterized by a febrile prodrome and convulsive SE. Clinical features and therapeutic response, which resemble those observed in inflammatory‐mediated disease, highlight the claustrum as involved in a widespread network dysfunction. Early recognition of this imaging feature may therefore assist in the diagnostic stratification of NORSE, inform therapeutic decision‐making, and prompt timely immunomodulatory intervention. Prospective, multicentre studies with standardized imaging protocols and longitudinal follow‐up are needed to clarify the claustrum sign's independent prognostic value and to better define its role in the pathophysiology of NORSE.

## Author Contributions

Margherita Burani: conceptualization (equal); data curation (lead); formal analysis (lead); writing – original draft (equal); writing – review and editing (equal); Stefano Meletti: conceptualization (lead); data curation (lead); formal analysis (lead); writing – original draft (equal); writing – review and editing (equal); Lorenzo Muccioli, Giada Giovannini, Niccolò Orlandi, Francesca Bisulli: data curation, review and editing (equal).

## Funding

The authors have nothing to report.

## Conflicts of Interest

S. Meletti received research grant support from the Ministry of Health (MOH); has received personal compensation as a scientific advisory board member for UCB, Jazz pharmaceuticals, and EISAI. F. Bisulli received research grant support from the Ministry of Health (MOH); has received personal compensation as a scientific advisory board member for UCB, Jazz pharmaceuticals, and Angelini Pharma. M. Burani, L. Muccioli, G. Giovannini, and N. Orlandi have no disclosures.

## Supporting information


**eMethods:** Extended literature search methodology.
**eTable1:** Characteristics of patients with claustrum sign.
**eTable2:** MRI alterations in patients without claustrum sign.
**eReference:** List of studies reporting NORSE cases without claustrum sign.
**eFigure1:** Worldwide distribution of the publications and of the reported cases.
**eFigure2:** Examples of claustrum sign from personal observations.

## Data Availability

The data that support the findings of this study are available from the corresponding author upon reasonable request.
