# Supplementary material for: Claustrum Involvement in New Onset Refractory Status Epilepticus: A Systematic Review
Source: Ann Clin Transl Neurol. 2026 Aug 4;13(9):1750–8. doi: 10.1002/acn3.70490 (PMC13438007; doi:10.1002/acn3.70490)
Supplement: Supplementary file 1 [file ACN3-13-1750-s001.docx]

**Claustrum involvement in New Onset Refractory Status Epilepticus: a systematic review**

Margherita Burani, Lorenzo Muccioli, Giada Giovannini, Niccolò Orlandi, Francesca Bisulli, Stefano Meletti.

**Supplementary Material**

**eMethods**……..page 2

**eTable1**……….page 3

**eTable2**……….page 10

**eReferences**…..page 12

**eFigure1**……....page 14

**eFigure2**………page 15

**eMethods**

The literature search was conducted on MEDLINE, Embase.com, and PubMed from 2018 to February 2026 using the following search string: “Febrile Infection-Related Epilepsy Syndrome” OR “FIRES” OR “NORSE” OR “New Onset Refractory Status Epilepticus” OR (“status epilepticus” AND “claustrum”) OR (“epilepsy” AND “claustrum”) OR (“status epilepticus” AND “neuroimaging”) OR (“NORSE” AND “neuroimaging”) OR (“NORSE” AND “MRI”).

**eTable1**. Characteristics of patients with claustrum sign

| **Study** | **Year** | **Patient** | **A/G** | **Time (d)** | **Other symptoms** | **SE type** | **ICU admission** | **Evolution of SE** | **Immunotherapy** | **Neurological outcome** | **Chronic Epilepsy** | **In-hospital death** | **Selection bias** | **Assessment bias** |
| --- | --- | --- | --- | --- | --- | --- | --- | --- | --- | --- | --- | --- | --- | --- |
| Bai | 2024 | 1 | 20/F | 7 | Fever | GCSE | Yes | na | IVMP, IVIG | na | Yes | No | High | High |
| Bai | 2024 | 2 | 21/M | 7 | Fever | GCSE | Yes | na | IVMP, IVIG, PLEX, CP | na | Yes | No | High | High |
| Bai | 2024 | 3 | 33/M | 19 | Fever | GCSE | No | na | IVMP, IVIG | na | No | No | High | High |
| Bai | 2024 | 4 | 9/F | 4 | Fever | GCSE | na | na | IVMP, IVIG | na | Yes | No | High | High |
| Bai | 2024 | 5 | 16/F | 5 | Fever | GCSE | Yes | na | IVMP, IVIG | na | No | No | High | High |
| Bai | 2024 | 6 | 26/M | 5 | Fever | GCSE | No | na | IVMP | na | No | No | High | High |
| Bai | 2024 | 7 | 23/F | 5 | Fever | GCSE | Yes | na | IVMP, IVIG | na | Yes | No | High | High |
| Bai | 2024 | 8 | 10/F | 5 | Fever | GCSE | Yes | na | IVMP, IVIG, TCZ | na | Yes | No | High | High |
| Bai | 2024 | 9 | 32/M | 5 | Fever | GCSE | Yes | na | IVMP, IVIG, RTX | na | Yes | No | High | High |
| Bai | 2024 | 10 | 9/F | 4 | Fever | GCSE | Yes | na | IVMP, IVIG | na | Yes | No | High | High |
| Bai | 2024 | 11 | 42/F | 5 | Fever | GCSE | No | na | IVMP | na | No | No | High | High |
| Bai | 2024 | 12 | 25/M | 5 | Fever | GCSE | Yes | na | IVMP, IVIG, PLEX | na | Yes | No | High | High |
| Bai | 2024 | 13 | 64/F | 7 | Fever | GCSE | No | na | IVMP | na | No | No | High | High |
| Bai | 2024 | 14 | 15/F | 7 | Fever | GCSE | No | na | IVMP, TCZ | na | No | No | High | High |
| Bai | 2024 | 15 | 20/M | 6 | Fever | GCSE | No | na | IVMP, IVIG | na | Yes | No | High | High |
| Bai | 2024 | 16 | 37/F | 7 | Fever | GCSE | Yes | na | IVMP | na | Yes | No | High | High |
| Bai | 2024 | 17 | 42/F | na | Fever | GCSE | No | na | IVMP | na | Yes | No | High | High |
| Bai | 2024 | 18 | 9/F | 8 | Fever | GCSE | na | na | IVMP, IVIG | na | No | No | High | High |
| Bai | 2024 | 19 | 17/M | 3 | Fever | GCSE | Yes | na | IVMP, IVIG | na | No | No | High | High |
| Bai | 2024 | 20 | 15/F | 3 | Fever | GCSE | No | na | IVMP | na | Yes | No | High | High |
| Basso | 2022 | 21 | 5/M | 1 | Fever, somnolence | Focal motor | Yes | RSE | IVMP, IVIG, PLEX | Mild aphasia | Yes | No | High | High |
| Di Dier | 2023 | 22 | 39/F | na | Confusion, hallucinations | Focal Myoclonic | na | na | na | na | na | No | High | High |
| Guo | 2023 | 23 | 19/F | 5 | Fever, flu, drowsiness, memory deficit | Focal to bilateral tonic-clonic SE | No | Responsive | IVMP | Normal | Yes | No | High | High |
| Muccioli | 2022 | 24 | 40/F | 6 | Fever, encephalopathy | na | Yes | SRSE | na | na | na | No | High | High |
| Riedel | 2025 | 25 | 23/F | na | Fever, URI | GCSE | Yes | RSE | IVMP | Mild attention and language deficit | No | No | High | High |
| Safan | 2023 | 26 | 30/M | 9 | Fever, headache, neck stiffness, delusions, echolalia | GCSE | Yes | RSE | IVMP, IVIG | Normal | No | No | High | High |
| Silva | 2019 | 27 | 16/F | 21 | Fever, memory loss, impaired consciousness | GCSE | No | na | na | Normal | No | No | High | High |
| Zhao | 2025 | 28 | 4/M | na | Fever, impaired consciousness | na | na | RSE | IVMP, IVIG | Memory and calculation deficit | Yes | No | High | High |
| Zhao | 2025 | 29 | 8/F | na | Fever, impaired consciousness, psychiatric symptoms | na | na | RSE | IVMP, IVIG, RTX, TCZ | Memory and calculation deficit | Yes | No | High | High |
| Zhao | 2025 | 30 | 11/M | na | Fever, impaired consciousness | na | na | RSE | IVMP, IVIG | Memory and calculation deficit | Yes | No | High | High |
| Zhao | 2025 | 31 | 4/M | na | Fever, impaired consciousness | na | na | RSE | IVMP, IVIG, PLEX, TCZ, Anakinra | Memory and calculation deficit | Yes | No | High | High |
| Zhao | 2025 | 32 | 7/F | na | Fever, impaired consciousness | na | na | RSE | IVMP, IVIG | Vegetative state | Yes | Vegetative state | High | High |
| Zhao | 2025 | 33 | 11/F | na | Fever, impaired consciousness, psychiatric symptoms | na | na | RSE | IVMP, IVIG, TCZ | Memory and calculation deficit | Yes | No | High | High |
| Zhao | 2025 | 34 | 8/M | na | Fever, impaired consciousness, psychiatric symptoms | na | na | RSE | IVMP, IVIG | Memory and calculation deficit | Yes | No | High | High |
| Zhao | 2025 | 35 | 4/M | na | Fever, impaired consciousness, psychiatric symptoms | na | na | RSE | IVMP, IVIG, RTX, TCZ | Normal | Yes | No | High | High |
| Zhao | 2025 | 36 | 5/M | na | Fever, impaired consciousness, psychiatric symptoms | na | na | RSE | IVMP, IVIG, TCZ | Normal | Yes | No | High | High |
| Zhao | 2025 | 37 | 6/F | na | Fever, impaired consciousness, psychiatric symptoms, speech dysfunction | na | na | RSE | IVMP, IVIG, TCZ, MMF | Memory and calculation deficit | Yes | No | High | High |
| Zhao | 2025 | 38 | 5/M | na | Fever, impaired consciousness, psychiatric symptoms | na | na | RSE | IVMP, IVIG | Memory and calculation deficit | Yes | No | High | High |
| Zhao | 2025 | 39 | 6/M | na | Fever, impaired consciousness, psychiatric symptoms | na | na | Responsive | IVMP, IVIG, PLEX, RTX; TCZ, MMF, Anakinra | Memory and calculation deficit | Yes | No | High | High |
| Choi | 2019 | 40 | 29/M | 10 | Fever, headache, myalgia, memory impairment | Focal to bilateral tonic-clonic SE | Yes | RSE | IVMP, IVIG | na | na | No | High | High |
| Choi | 2019 | 41 | 52/F | 3 | Fever, URI, confusion, memory impairment | GCSE | Yes | SRSE | IVMP, IVIG | na | na | No | High | High |
| Choi | 2019 | 42 | 59/F | 5 | Fever, myalgia, headache, memory and language impairment, somnolence | Focal to bilateral tonic-clonic SE | Yes | RSE | IVMP, IVIG, RTX | na | na | No | High | High |
| Choi | 2019 | 43 | 51/M | na | Myalgia, URI, memory impairment | GCSE | Yes | SRSE | IVMP, IVIG, PLEX | na | na | No | High | High |
| Choi | 2019 | 44 | 39/F | 7 | Fever, nausea, memory impairment | GCSE | Yes | SRSE | IVMP, IVIG, PLEX | na | na | Yes | High | High |
| Choi | 2019 | 45 | 34/M | 7 | Fever, myalgia, somnolence, confusion, language impairment | GCSE | Yes | SRSE | IVMP, IVIG, PLEX | na | na |  | High | High |
| Choi | 2019 | 46 | 30/F | na | Headache, confusion, language impairment | GCSE | Yes | SRSE | IVMP, IVIG | na | na | Yes | High | High |
| Choi | 2019 | 47 | 46/M | 4 | Fever, headache, myalgia, memory and language impairment | Focal to bilateral tonic-clonic SE | Yes | SRSE | IVMP, IVIG | na | na | Yes | High | High |
| Choi | 2019 | 48 | 46/M | 6 | Fever, headache, somnolence, confusion, labile mood | Focal to bilateral tonic-clonic SE | Yes | SRSE | IVMP, IVIG, RTX | na | na | No | High | High |
| Choi | 2019 | 49 | 32/M | 8 | Fever, Myalgia, URI, confusion | GCSE | Yes | SRSE | IVMP, IVIG, PLEX | na | na | No | High | High |
| Ungureanu | 2019 | 50 | 62/M | 3 | Fever, psychosis | Focal to bilateral tonic-clonic SE | Yes | SRSE | IVMP | Normal | No | No | High | High |
| Yang | 2021 | 51 | 16/F | na | Fever, agitation, garrulousness | na | No | na | No | Memory loss | Yes | No | High | High |
| Yang | 2021 | 52 | 26/F | na | Fever, headache, agitation, language disturbances | na | No | na | No | Memory loss | Yes | No | High | High |
| Yang | 2021 | 53 | 29/F | na | Fever, headache, agitation, language disturbances | na | No | na | No | Normal | Yes | No | High | High |
| Yang | 2021 | 54 | 20/F | na | Fever | GCSE | Yes | SRSE | No | Memory loss | Yes | No | High | High |
| Yang | 2021 | 55 | 23/F | na | Fever agitation | Focal motor | No | RSE | No | Memory loss | Yes | No | High | High |
| Silva | 2018 | 56 | 6/F | 7 | Fever, headache, anorexia | Focal to bilateral tonic-clonic SE | Yes | RSE | IVMP, IVIG | Normal | No | No | High | High |
| Konomatsu | 2025 | 57 | 24/F | 4 | Fever, gastric discomfort | Focal to bilateral tonic-clonic SE | No | RSE | IVMP | Normal | Yes | No | High | High |
| Abdullah | 2025 | 58 | 42/M | na | na | na | Yes | SRSE | IVMP, IVIG, PLEX, RTX, CP | na | na | No | High | High |
| Steriade | 2018 | 59 | 36/M | na | Fever and encephalopathy | GCSE | Yes | SRSE | IVMP, IVIG, PLEX | Normal | Yes | No | High | High |
| Steriade | 2018 | 60 | 25/M | na | Fever | GCSE | Yes | SRSE | IVMP, IVIG | Normal | Yes | No | High | High |
| Yorichika | 2024 | 61 | 44/F | na | na | GCSE | Yes | SRSE | IVMP, IVIG, TCZ, CP | na | Yes | No | High | High |
| Tan | 2026 | 62 | 21/F | 7 | Fever, URI | GCSE | Yes | SRSE | IVMP, PLEX | na | na | Yes | High | High |
| Saad | 2025 | 63 | 3/M | na | na | Focal motor | Yes | na | Yes (na) | na | Yes | No | High | High |
| Kim | 2020 | 64 | 52/M | na | Fever | NCSE | na | RSE | na | na | No | No | High | High |
| Kim | 2020 | 65 | 21/M | na | Fever | GCSE | na | SRSE | Yes (na) | na | Yes | No | High | High |
| Kim | 2020 | 66 | 40/M | na | Fever | Focal motor | na | SRSE | Yes (na) | na | Yes | No | High | High |
| Caporale | 2025 | 67 | 10/F | 5 | Fever, cacosmia, dysphagia, drowsiness | Focal to bilateral tonic-clonic SE | Yes | SRSE | IVMP, IVIG, Anakinra | Mild cognitive impairment | Yes | No | High | High |
| Acampora | 2022 | 68 | 23/M | 14 | Fever, myalgia, asthenia | GCSE | Yes | RSE | IVMP, IVIG | Normal | Yes | No | High | High |
| Mizutani | 2019 | 69 | 30/M | 7 | Fever, diarrhoea, headache | GCSE | Yes | SRSE | IVMP, IVIG, PLEX | na | na | No | High | High |
| Muccioli | 2026 | 70 | 32/F | 6 | Fever | GCSE | Yes | SRSE | na | na | na | Yes | High | High |
| Kumar | 2024 | 71 | 30/F | na | Fever, headache, behavioural chianges | GCSE | na | SRSE |  | na | na | No | High | High |

A/G: age/gender; d: days; ICU: Intensive Care Unit; SE: Status Epilepticus; F: female; M: male; URI: upper respiratory dysfunction; GCSE: Generalized Convulsive Status Epilepticus; RSE: Refractory Status Epilepticus; SRSE: Super Refractory Status Epilepticus; IVMP: intravenous methylprednisolone; IVIG: intravenous immunoglobulin; PLEX: plasma exchange; RTX: Rituximab; CP: cyclophosphamide; TCZ: Tocilizumab; MMF: Mycophenolate Mofetil; na: not available.

**eTable2.** MRI alterations in patients without claustrum sign.

| **Study** | **N of patients** | **MRI findings** |
| --- | --- | --- |
| Astner-Rohracher 2023 | 1 | diffusion restriction and FLAIR hyperintensity in the right temporo-occipital region |
| Ruttkowski 2024 | 1 | diffusion restriction in the basal ganglia |
| Haanpää 2022 | 13 | bilateral MTL and bilateral thalamus FLAIR hyperintensity; head of caudatus and putamen FLAIR hyperintensity; bilateral frontal temporal and parietal lobes FLAIR hyperintensity; 10 normals. |
| Yorichika 2025 | 3 | bilateral MTL FLAIR hyperintensity |
| Saad 2025 | 18 | bilateral MTL, basal ganglia and/or insula FLAIR hyperintensity; 12 normals |
| Lee 2018 | 29 | leptomeningeal enhancement; MTL and thalamus FLAIR hyperintensity; 16 normals |
| Fan 2025 | 5 | diffuse cortical alterations; frontal, basal ganglia and insular FLAIR hyperintensity; putamen FLAIR hyperintensity; 2 normals |
| Shi 2023 | 11 | bilateral cingulate gyrus, fronto-temporal and insular FLAIR hyperintensity; corpus callosum FLAIR hyperintensity; MTL FLAIR hyperintensity; 8 normals |
| Aurangzeb 2020 | 3 | bilateral MTL and basal ganglia FLAIR hyperintensity |
| Ochiai 2025 | 1 | bilateral MTL FLAIR hyperintensity |
| Watanabe 2023 | 1 | bilateral MTL FLAIR hyperintensity |
| Meenakshi-Sundaram 2021 | 1 | bilateral MTL FLAIR hyperintensity |
| Kim 2020 | 36 | leptomeningeal enhancement; MTL and sulcus FLAIR hyperintensity; hippocampal atrophy; 11 normals |
| Dilena 2019 | 1 | temporo-occipital and insular FLAIR hyperintensity |
| Murugesu 2025 | 7 | diffuse cortical and subcortical alterations; bilateral MTL FLAIR hyperintensity; 3 normals |
| Choi 2019 | 3 | bilateral MTL FLAIR hyperintensity |

MRI: Magnetic Resonance Imaging; FLAIR: Fluid-Attenuated Inversion Recovery; MTL: Medial Temporal Lobe.

**eReference**

1. Astner-Rohracher A, Mauritz M, Leitinger M, Rossini F, Kalss G, Neuray C, Retter E, Wortmann SB, Achleitner MT, Mayr JA, Trinka E. A case report: New-onset refractory status epilepticus in a patient with *FASTKD2*-related mitochondrial disease. Front Neurol. 2023 Jan 11;13:1063733. doi: 10.3389/fneur.2022.1063733. PMID: 36712458; PMCID: PMC9875587.
2. Ruttkowski L, Wallot I, Korell M, Daur E, Seipelt P, Leonhardt A, Weber S, Mand N. Case report: Febrile infection-related epilepsy syndrome in a 14-year-old girl with multiple organ failure and lethal outcome. Front Neurosci. 2024 Mar 5;18:1255841. doi: 10.3389/fnins.2024.1255841. PMID: 38505775; PMCID: PMC10948546.
3. Haanpää A, Laakso SM, Kinnunen A, Kämppi L, Forss N. Early clinical features of new-onset refractory status epilepticus (NORSE) in adults. BMC Neurol. 2022 Dec 20;22(1):495. doi: 10.1186/s12883-022-03028-y. PMID: 36539824; PMCID: PMC9764533.
4. Yorichika Y, Neshige S, Sakahara H, Ono N, Nonaka M, Tagane Y, Watanabe T, Tachiyama K, Ishibashi H, Nakamori M, Shishido T, Aoki S, Ueno H, Yamazaki Y, Iizuka T, Maruyama H. Early initiation of intravenous cyclophosphamide and one-year outcome in super-refractory cryptogenic-new onset refractory status epilepticus. Epilepsia Open. 2025 Feb;10(1):307-313. doi: 10.1002/epi4.13055. Epub 2024 Dec 19. PMID: 39701580; PMCID: PMC11803287.
5. Saad K, Aboelgheet AM, Hamed Y, Alruwaili TAM, Ibrahim MFM, Elhoufey A, Dailah HG, Taha SA, Al-Atram AA, Taha S, Abu-Elnaga O, Hassan AE, Gad EF. Febrile Infection-Related Epilepsy Syndrome (FIRES) in children: a retrospective cohort study. Eur J Pediatr. 2025 Feb 24;184(3):206. doi: 10.1007/s00431-025-06047-2. PMID: 39988600.
6. Lee HF, Chi CS. Febrile infection-related epilepsy syndrome (FIRES): therapeutic complications, long-term neurological and neuroimaging follow-up. Seizure. 2018 Mar;56:53-59. doi: 10.1016/j.seizure.2018.02.003. Epub 2018 Feb 6. PMID: 29453111.
7. Fan M, Li Y, Diao H, Wang Y. Ketogenic Diet Therapy for Adult Febrile Infection-Related Epilepsy Syndrome (FIRES): A Case Series of Five Patients and Clinical Efficacy Analysis. Infect Drug Resist. 2025 Dec 24;18:6875-6888. doi: 10.2147/IDR.S574683. PMID: 41466766; PMCID: PMC12744823.
8. Shi X, Wang Y, Wang X, Kang X, Yang F, Yuan F, Jiang W. Long-term outcomes of adult cryptogenic febrile infection-related epilepsy syndrome (FIRES). Front Neurol. 2023 Jan 4;13:1081388. doi: 10.3389/fneur.2022.1081388. PMID: 36686522; PMCID: PMC9848432.
9. Aurangzeb S, Prisco L, Adcock J, Speirs M, Raby S, Westbrook J, Sen A. New-onset super refractory status epilepticus: A case-series. Seizure. 2020 Feb;75:174-184. doi: 10.1016/j.seizure.2019.10.005. Epub 2019 Nov 9. PMID: 31757748.
10. Ochiai K, Omata T, Sano K, Sakuma H, Takanashi JI. Potential Role of Perampanel in Reducing Barbiturate Dependency in Febrile Infection-Related Epilepsy Syndrome: A Case Report. Cureus. 2025 Jan 16;17(1):e77568. doi: 10.7759/cureus.77568. PMID: 39958143; PMCID: PMC11830119.
11. Watanabe T, Nakamori M, Ishikawa K, Motoda A, Neshige S, Yamamoto S, Katahira K, Higaki T, Ueno H, Iizuka T, Maruyama H. Reversible Brain Atrophy in Cryptogenic New-onset Refractory Status Epilepticus. Intern Med. 2023 Jun 15;62(12):1835-1842. doi: 10.2169/internalmedicine.0465-22. Epub 2022 Nov 9. PMID: 36351577; PMCID: PMC10332979.
12. Meenakshi-Sundaram S, Sankaranarayanan M, Jeyaraman M, Ayyappan C, Karthik SN, Pandi S. Super refractory status in a case of Febrile Infection-Related Epilepsy Syndrome due to hemophagocytic lymphocytic histiocytosis. Epilepsia Open. 2021 Jan 15;6(1):22-27. doi: 10.1002/epi4.12454. PMID: 33681644; PMCID: PMC7918320.
13. Kim HJ, Lee SA, Kim HW, Kim SJ, Jeon SB, Koo YS. The timelines of MRI findings related to outcomes in adult patients with new-onset refractory status epilepticus. Epilepsia. 2020 Aug;61(8):1735-1748. doi: 10.1111/epi.16620. Epub 2020 Jul 27. PMID: 32715470.
14. Dilena R, Mauri E, Aronica E, Bernasconi P, Bana C, Cappelletti C, Carrabba G, Ferrero S, Giorda R, Guez S, Scalia Catenacci S, Triulzi F, Barbieri S, Calderini E, Vezzani A. Therapeutic effect of Anakinra in the relapsing chronic phase of febrile infection-related epilepsy syndrome. Epilepsia Open. 2019 Mar 27;4(2):344-350. doi: 10.1002/epi4.12317. PMID: 31168503; PMCID: PMC6546072.
15. Murugesu S, Mohamed AR, Musa HB, Lee JX, Anuar MA, Khoo TB. Tocilizumab for super-refractory status epilepticus in children with FIRES: A case series. Seizure. 2026 Jan;134:180-185. doi: 10.1016/j.seizure.2025.11.019. Epub 2025 Nov 28. PMID: 41401655.
16. Choi JY, Kim EJ, Moon SY, Kim TJ, Huh K. Prognostic significance of subsequent extra-temporal involvement in cryptogenic new onset refractory status epilepticus (NORSE) initially diagnosed with limbic encephalitis. Epilepsy Res. 2019 Dec;158:106215. doi: 10.1016/j.eplepsyres.2019.106215. Epub 2019 Oct 12. PMID: 31669912.

**eFigure1: Worldwide distribution of the publications and of the reported cases.**

Red circles denote cases with the claustrum sign, while blue circles represent cases without claustral abnormalities. Circle size is proportional to the number of cases.

Groups

With claustrum sign

Without claustrum sign

**eFigure2: Examples of claustrum sign from personal observations.**

We report 6 case/examples of axial/coronal FLAIR – T2 sequences showing typical claustrum hyperintensity.
